# Mutualist-Provisioned Resources Impact Vector Competency

**DOI:** 10.1128/mBio.00018-19

**Published:** 2019-06-04

**Authors:** Rita V. M. Rio, Anna K. S. Jozwick, Amy F. Savage, Afsoon Sabet, Aurelien Vigneron, Yineng Wu, Serap Aksoy, Brian L. Weiss

**Affiliations:** a Department of Biology, Eberly College of Arts and Sciences, West Virginia University, Morgantown, West Virginia, USA; b Department of Epidemiology of Microbial Diseases, Yale School of Public Health, Yale University, New Haven, Connecticut, USA; University of Texas at Austin

**Keywords:** *Wigglesworthia*, folate, trypanosome, tsetse, vector competence

## Abstract

Many symbionts supplement their host’s diet with essential nutrients. However, whether these nutrients also enhance parasitism is unknown. In this study, we investigated whether folate (vitamin B_9_) production by the tsetse fly (*Glossina* spp.) essential mutualist, *Wigglesworthia*, aids auxotrophic African trypanosomes in completing their life cycle within this obligate vector. We show that the expression of *Wigglesworthia* folate biosynthesis genes changes with the progression of trypanosome infection within tsetse. The disruption of *Wigglesworthia* folate production caused a reduction in the percentage of flies that housed midgut (MG) trypanosome infections. However, decreased folate did not prevent MG trypanosomes from migrating to and establishing an infection in the fly’s salivary glands, thus suggesting that nutrient requirements vary throughout the trypanosome life cycle. We further substantiated that trypanosomes rely on symbiont-generated folate by feeding this vitamin to Glossina brevipalpis, which exhibits low trypanosome vector competency and houses *Wigglesworthia* incapable of producing folate. Folate-supplemented G. brevipalpis flies were significantly more susceptible to trypanosome infection, further demonstrating that this vitamin facilitates parasite infection establishment. Our cumulative results provide evidence that *Wigglesworthia* provides a key metabolite (folate) that is “hijacked” by trypanosomes to enhance their infectivity, thus indirectly impacting tsetse species vector competency. Parasite dependence on symbiont-derived micronutrients, which likely also occurs in other arthropod vectors, represents a relationship that may be exploited to reduce disease transmission.

## Abstract (precis)

Parasites elicit several physiological changes in their host to enhance transmission. Little is known about the functional association between parasitism and microbiota-provisioned resources typically dedicated to animal hosts and how these goods may be rerouted to optimize parasite development. This study is the first to identify a specific symbiont-generated metabolite that impacts insect vector competence by facilitating parasite establishment and, thus, eventual transmission. Specifically, we demonstrate that the tsetse fly obligate mutualist *Wigglesworthia* provisions folate (vitamin B_9_) that pathogenic African trypanosomes exploit in an effort to successfully establish an infection in the vector’s MG. This process is essential for the parasite to complete its life cycle and be transmitted to a new vertebrate host. Disrupting metabolic contributions provided by the microbiota of arthropod disease vectors may fuel future innovative control strategies while also offering minimal nontarget effects.

## INTRODUCTION

Animals host a spectrum of microbes ranging from beneficial symbionts to detrimental parasites. Mutualists enhance their host’s health, as evidenced by higher fitness when living jointly than when living apart. In contrast, a classical hallmark of parasitism involves the exploitation of resources within a host without any reciprocation (1). To date, little is known about the impact of parasitism on mutualist-provisioned benefits and whether symbiont-provisioned goods may be exploited to optimize parasite development. A more complete understanding of these metabolic relationships will provide further insight into host-associated phenotypes, such as vector competency in the case of arthropods that transmit disease.

Tsetse flies (*Glossina* spp.) are of medical and veterinary significance because they serve as the obligate vector of pathogenic African trypanosomes (*Trypanosoma* spp.). These flagellated protozoa (Trypanosoma brucei subspp.) are the causative agents of human and animal African trypanosomiases (HAT and AAT, respectively) (2). These diseases significantly impact population demographics over a wide swath of sub-Saharan Africa because they inflict devastating public health and socioeconomic consequences by exacerbating disease burden and detrimentally impacting livestock-based agriculture.

While only a small proportion of tsetse flies are infected with trypanosomes, all individuals harbor a microbiota that consists of indigenous endosymbionts as well as a population of environmentally acquired enteric bacteria (3–6). Relative to the tsetse’s physiological homeostasis, the most significant member of this bacterial consortium is the obligate mutualist *Wigglesworthia* species (7). This bacterium is housed intracellularly in the tsetse’s midgut (MG)-associated bacteriome organ and extracellularly in maternal milk secretions (8, 9). *Wigglesworthia* and tsetse share a lengthy coevolutionary history that dates back 50 million to 80 million years (10). The antiquity of the tsetse-*Wigglesworthia* partnership is especially apparent upon examination of the bacterium’s genome, which has been purged of many genes deemed unnecessary for a strictly endosymbiotic lifestyle. However, despite this genome tailoring, *Wigglesworthia* retains the genetic inventory necessary to metabolically complement the tsetse’s strictly hematophagous diet (11–13). More specifically, *Wigglesworthia*’s chromosome encodes many cofactor biosynthesis pathways, including those of multiple B vitamins, although variation in biosynthetic abilities occurs between different bacterial isolates (14, 15). Correspondingly, elimination of *Wigglesworthia* via antibiotic treatment renders female tsetse reproductively sterile (16–20). This phenotype can be partially reversed by provisioning *Wigglesworthia*-free flies with B vitamins, yeast extract, and/or homogenized bacteriomes (13, 15, 20), thus supporting a critical role of this bacterium in the supplementation of nutrients that are either absent or present at low levels in vertebrate blood.

Comparative genomic analyses indicate that *Wigglesworthia* bacteria from different tsetse species exhibit few distinctions in their inventories of genes associated with tsetse metabolic complementation. This finding is indicative of high parallel natural selection of *Wigglesworthia* bacteria following the divergence of their respective tsetse host species (14, 15). However, specific *Wigglesworthia* isolates exhibit differences in their abilities to synthesize folate (vitamin B_9_) *de novo* (12, 15). Tsetse flies are folate auxotrophs and as such must acquire this vitamin from external (blood) and/or internal (*Wigglesworthia*) sources to serve as a cofactor in various enzymatic activities. B_9_ provisioning is critical to fly reproduction, with enzymatic inhibition of symbiont production curtailing reproductive output (12). Interestingly, trypanosomes are also unable to make folate (21), and they circumvent this metabolic deficiency by using multiple transporters to salvage the vitamin from their environment (22, 23). These metabolites are incorporated into parasite C-1 metabolic pathways, which are crucial for various cellular events, including genome modifications (e.g., DNA methylation), and for the biosynthesis of nucleotides and multiple amino acids (24, 25).

To date, no studies have been performed to determine if and how *Wigglesworthia* folate biosynthesis and allocation within tsetse are impacted by trypanosome challenge or whether these processes facilitate the establishment of infections and, thus, fly vector competence (defined as the ability of an arthropod vector to acquire, maintain, and transmit a pathogen [26]). Intriguingly, tsetse fly species that harbor isolates of *Wigglesworthia* incapable of producing folate exhibit a concomitant reduction in trypanosome infection susceptibility (27–31). This natural variation in metabolic capacity implies that an interdependence exists between symbiont biosynthesis and the provisioning of folate and trypanosome infection establishment (and, thus, tsetse vector competency). To experimentally address this hypothesis, we investigated the relationship between *Wigglesworthia* folate biosynthesis and provisioning and the susceptibility of tsetse to trypanosome infections. We first characterize the impact of trypanosome challenge and infection maturation on *Wigglesworthia* folate biosynthetic metabolism. We then describe how disruption of *Wigglesworthia* folate production impacts the establishment of trypanosome infections in the tsetse’s MG and salivary glands (SGs). Additionally, we investigate whether a normally trypanosome-refractory tsetse species, which harbors *Wigglesworthia* bacteria that lack a functional folate biosynthesis pathway, can be made more susceptible to infection by supplementing the flies’ diet with exogenous folate. The work described here provides further insight into the importance of symbiont metabolic provisioning for tsetse species and, additionally, its importance for trypanosome establishment and persistence.

## RESULTS

### The expression of Wigglesworthia morsitans chorismate and folate biosynthesis loci during the progression of trypanosome infections.

The impact of Trypanosoma brucei *brucei* strain RUMP 503 infection on the expression of Wigglesworthia morsitans chorismate and folate biosynthetic loci was examined in female and male flies 2 weeks (early stage [ES]) and 5 weeks (late stage [LS]) after parasite challenge. Following dissection to monitor for the presence or absence of parasites, these flies were designated either infected (IN) (parasites were observed in the MG or in the MG and SGs of tsetse harboring ES or LS infections, respectively) or parasite refractory (PR) (meaning that trypanosome-challenged tsetse flies were able to clear parasites). Age-matched nonchallenged (NC) individuals were used as controls. We monitored the expression of genes that encode proteins associated with folate biosynthesis pathways (i.e., shikimate pathway and the pABA [*para*-aminobenzoate] and pterin biosynthesis branches) at these two time points. More specifically, we examined the transcriptional profile of the genes that encode 3-phosphoshikimate 1-carboxyvinyltransferase (*aroA*), which catalyzes the sixth step in chorismate production (shikimate pathway), and *para*-aminobenzoate synthetase component I (*pabB*) and 7,8-dihydropteroate synthase (*folP*), which encode proteins involved in the first step of the pABA biosynthesis branch and the first step of the conversion of chorismate to folate, respectively (see Fig. S1 in the supplemental material).

10.1128/mBio.00018-19.1 FIG S1 The *Wigglesworthia* isolate harbored within *G. morsitans* (*W. morsitans*) retains the complete chorismate and folate biosynthesis pathways. The sequential chorismate and folate production pathways are represented by arrows indicating steps catalyzed by the indicated enzymes. The initial molecule phosphoenolpyruvate (PEP) is a byproduct of glycolysis, while erythrose-4-phosphate is an intermediate in the pentose phosphate pathway. The enzymatic inhibition of glyphosate toward AroA is shown. Download FIG S1, PDF file, 0.5 MB. Copyright © 2019 Rio et al. 2019 Rio et al. This content is distributed under the terms of the Creative Commons Attribution 4.0 International license.

**FIG 1:**
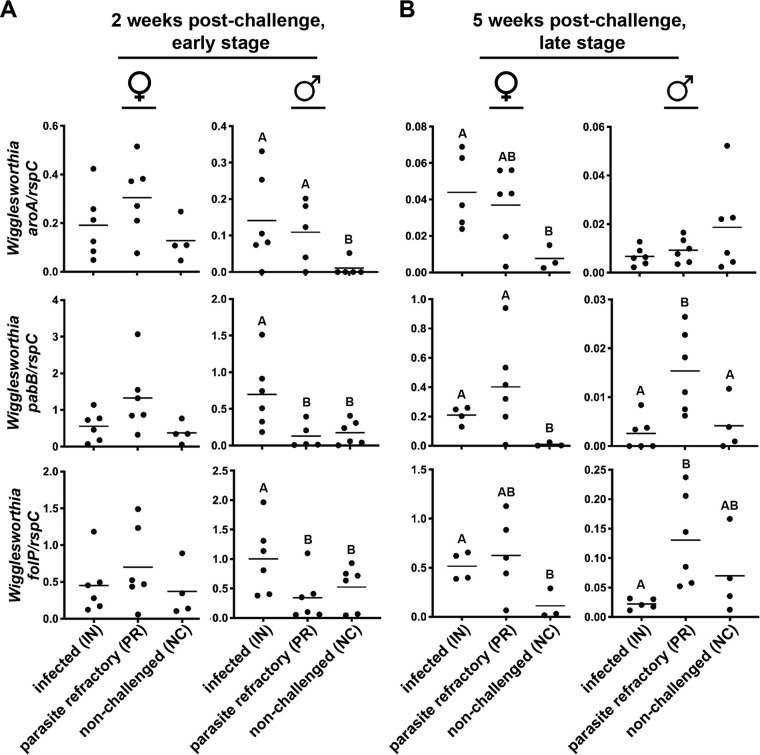
*W. morsitans* chorismate and folate biosynthetic locus expression from the bacteriomes from early-stage (ES) (2 weeks) (A) and late-stage (LS) (5 weeks) (B) *T. b. brucei* RUMP 503 challenges within virgin female and male tsetse. *W. morsitans* chorismate and folate biosynthetic locus expression levels were normalized to the *W. morsitans rpsC* expression level. Infection status is indicated. Locus expression levels were compared using one-way ANOVA followed by a Tukey-Kramer *post hoc* pairwise comparison of the mean. Different letters represent statistical significance between groups (*P ≤ *0.05), with each dot representing data for an individual fly. Horizontal lines represent the means.

We observed no statistically significant difference in the transcriptional activities of any of the three W. morsitans loci during the ES in the bacteriomes of females regardless of infection status (IN, PR, or NC) (Fig. 1A). In contrast, *W. morsitans* within the bacteriomes of IN and PR males at the ES expressed significantly higher levels of *aroA* than did *W. morsitans* from age-matched NC flies (*P < *0.0001 by Tukey’s multiple-comparison test). Additionally, during ES, *W. morsitans* within the bacteriomes of IN males expressed significantly more *pabB* (*P < *0.0001 by Tukey’s multiple-comparison test) and *folP* (*P = *0.0006 by Tukey’s multiple-comparison test) than did their age-matched PR and NC counterparts (Fig. 1A). During the LS, *W. morsitans* residing within IN and PR females expressed higher levels of *aroA*, *pabB*, and *folP* than in age-matched NC females, with statistical significance in all three loci observed between the IN and NC females (*P < *0.05 by Tukey’s multiple-comparison test) and within PR and NC females for *pabB* (*P = *0.03 by Tukey’s multiple-comparison test) (Fig. 1B). In males, *aroA* transcript abundance was not significantly different during the LS among any of the three groups. However, during the LS, *W. morsitans* from PR males expressed significantly more *pabB* transcripts than did *W. morsitans* in IN and NC individuals (*P < *0.05 by Tukey’s multiple-comparison test), and *W. morsitans* from PR and NC males expressed more *folP* transcripts than did *W. morsitans* from age-matched IN individuals, with statistical significance observed between PR and IN males (Fig. 1B) (*P = *0.019 by Tukey’s multiple-comparison test). Finally, higher expression levels for *W. morsitans aroA*, *pabB*, and *folP* were generally observed within the bacteriomes of ES flies than in their LS counterparts of both sexes, although statistical significance was observed only between PR females for *aroA* and *pabB* expression (with 7- and 3-fold-higher transcript abundances, respectively [*P ≤ *0.01 by Tukey’s multiple-comparison test]) and between IN males for all three loci examined (with 20- to 200-fold-higher transcript abundances [*P ≤ *0.002 by Tukey’s multiple-comparison test]). This outcome indicates that, in addition to host nutritional demands fluctuating during development and aging, the expression of *W. morsitans* folate biosynthesis genes appears to be impacted by tsetse sex and the progression of trypanosome infections and that the demand is greater for the gene products during ES than during LS trypanosome challenge within tsetse.

### *Wigglesworthia*-derived folate modulates trypanosome infection outcomes.

Tsetse and trypanosomatids, including members of the genus *Trypanosoma*, are folate auxotrophs and thus must obtain this essential metabolite from their extracellular environment in order to survive (32, 33). In the tsetse model system, probable sources of exogenous folate include vertebrate blood (34, 35) and symbiotic *Wigglesworthia* (12). Because all tsetse feed on vertebrate blood, the availability of additional folate, derived from *Wigglesworthia*, may be a factor that determines whether or not African trypanosomes are able to successfully infect a tsetse host. To test this theory, we inhibited *W. morsitans* folate production *in vivo* by feeding Glossina morsitans flies a blood meal supplemented with 100 μM glyphosate [*N-*(phosphonomethyl)glycine]. Glyphosate enzymatically inhibits AroA activity by competitively blocking phosphoenolpyruvate (PEP) binding and, thus, the downstream chorismate biosynthesis pathway that leads to folate production (36–38). When added to the tsetse blood meal, glyphosate inhibits the expression of *W. morsitans* folate biosynthesis loci, which decreases folate abundance within the fly’s bacteriome (12). Here we verified that 100 μM glyphosate interferes with the *W. morsitans* folate production pathway within bacteriomes, as supported by the reduced expression of *aroC* in treated flies (Fig. S2A). Furthermore, *Wigglesworthia* titers are unaffected following glyphosate treatment, and this inhibition is likely specific to the folate biosynthesis pathway because other critical pathways (i.e., *thiC* involved in B_1_ synthesis) are not disrupted (Fig. S2B).

10.1128/mBio.00018-19.2 FIG S2 (A) Expression of *W. morsitans aroC*, which catalyzes the seventh step in chorismate production, within bacteriomes following glyphosate supplementation in tsetse blood meals. The expression of *W. morsitans aroC*, located immediately downstream of *aroA*, significantly decreases with glyphosate supplementation of blood meals relative to the control. *W. morsitans rpsC* was used as a reference gene for normalization. The expression of *aroC*-*rpsC* in untreated age-matched tsetse was set to a value of 1. Sample sizes (*n*) are indicated. (B) *W. morsitans* (*Wgm*) titers and *thiC* expression (involved in B_1_ synthesis) remain unaffected following glyphosate treatment. Each dot represents *W. morsitans* expression within the bacteriome of an individual fly. Download FIG S2, PDF file, 0.2 MB. Copyright © 2019 Rio et al. 2019 Rio et al. This content is distributed under the terms of the Creative Commons Attribution 4.0 International license.

**FIG 2:**
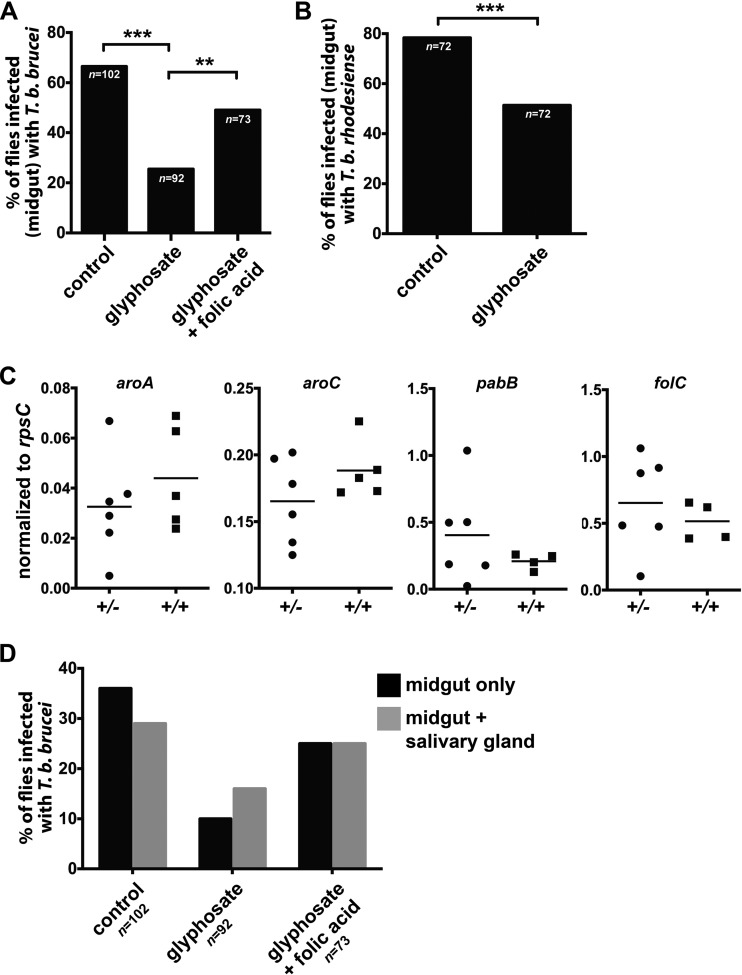
(A) Absolute *G. morsitans* MG infection rates (percent) during the ES in flies challenged with *T. b. brucei* RUMP 503. Control, flies maintained on blood only; glyphosate, flies maintained on blood plus 100 μM glyphosate; glyphosate + folic acid, flies maintained on blood plus 100 μM glyphosate and 500 nM folic acid. The means from three independent trials are indicated. Statistical significance is indicated with asterisks (**, *P ≤ *0.01; ***P ≤ 0.001). (B) Absolute *G. morsitans* MG infection rates (percent) during the ES in flies challenged with *T. b. rhodesiense* YTat 1.1. Control, flies maintained on blood only; glyphosate, flies maintained on blood plus 100 μM glyphosate. The means from three independent trials are indicated. Samples sizes (*n*) are provided. Statistical significance is indicated with asterisks (***, *P ≤ *0.001). (C) *W. morsitans* chorismate and folate biosynthetic locus expression from the bacteriomes of LS *T. b. brucei* RUMP 503-infected tsetse. *W. morsitans* chorismate and folate biosynthetic locus expression levels were normalized to the *W. morsitans rpsC* expression level. Infection status is indicated with +/−, indicating a nonpermissive infection, and +/+, indicating a permissive infection, representing trypanosomes harbored in the MG only and trypanosomes found in MG and SGs, respectively. Locus expression levels were compared using either Student’s *t* test or a Mann-Whitney test. Each point represents data for an individual fly. Horizontal lines represent the means. (D) Percentage of *T. b. brucei-*infected flies with midgut or salivary gland infections in LS challenged *G. morsitans* flies. Control, flies maintained on blood only; glyphosate, flies maintained on blood plus 100 μM glyphosate; glyphosate + folic acid, flies maintained on blood plus 100 μM glyphosate and 500 nM folic acid. Samples sizes (*n*) are provided.

We next investigated whether *in vivo* inhibition of *W. morsitans* folate production (via supplementation of tsetse blood meals with exogenous glyphosate) impacted trypanosome infection outcomes in tsetse. For this experiment, teneral G. morsitans flies were fed a blood meal that contained 100 μM glyphosate and *T. b. brucei* RUMP 503. This concentration of glyphosate does not detrimentally impact trypanosome growth *in vitro* (Fig. S3), indicating that treatment with this chemical will not directly affect the ability of trypanosomes to successfully infect and replicate within treated flies. We observed that significantly fewer glyphosate-supplemented *G. morsitans* flies (26%) housed *T. b. brucei* MG infections than wild-type controls (67%) (Fig. 2A). We next generated an additional cohort of *G. morsitans* flies in which we complemented the folate-depleted environment in glyphosate-treated flies by also including folic acid (500 nM) in the blood meal. Forty-nine percent of individuals that received both glyphosate and folic acid harbored *T. b. brucei* in their MG, indicating that blood meal supplementation with this vitamin reverses the inhibitory effect of glyphosate (Fig. 2A). We next assessed whether a different, T. brucei subspecies, Trypanosoma brucei *rhodesiense*, would also exhibit a similar impediment toward MG establishment upon the inhibition of *W. morsitans* folate biosynthesis. To do so, two groups of teneral flies were administered *T. b. rhodesiense-*inoculated blood meals, one of which also contained glyphosate (100 μM). Glyphosate treatment again significantly inhibited parasite infection establishment, as we observed that 50% of glyphosate-treated flies housed *T. b. rhodesiense* in their MG, compared to 79% of nontreated controls (Fig. 2B). Notably, MG infection prevalence was higher following challenge with *T. b. rhodesiense* than following challenge with *T. b. brucei* in both the presence and absence of glyphosate (control, 79% versus 67% for *T. b. rhodesiense* and *T. b. brucei*, respectively; glyphosate treatment, 50% versus 26% for *T. b. rhodesiense* and *T. b. brucei*, respectively). It stands to reason that different parasite subspecies, or even strains within subspecies, vary in their capacities to acquire folate and/or impact symbiont folate metabolism, as this may be one factor that regulates the ability of distinct parasites to successfully infect tsetse.

10.1128/mBio.00018-19.3 FIG S3 Bloodstream *T. b. brucei* RUMP 503 doubling rate with or without glyphosate within *in vitro* culture. With 100 μM glyphosate supplementation, neither trypanosome growth nor morphology was affected. The means and standard errors of the means (SEM) from two independent trials are demonstrated. Download FIG S3, PDF file, 0.04 MB. Copyright © 2019 Rio et al. 2019 Rio et al. This content is distributed under the terms of the Creative Commons Attribution 4.0 International license.

The trypanosome extrinsic incubation period (EIP) begins when tsetse consume a blood meal containing bloodstream-form parasites and culminates when vertebrate-infectious SG metacyclic trypomastigotes are transmitted to a naive host during a subsequent feeding (39). However, not all trypanosomes that successfully infect the tsetse’s MG go on to also infect the fly’s SGs and, thus, become transmissible (40). This outcome is likely reflective of the tsetse’s robust immune system, which is comprised of passive and active barriers that trypanosomes must circumvent in order to migrate from the fly’s MG to its SGs (8, 40). Hence, we were interested in examining whether *W. morsitans* folate biosynthesis may differ between flies that harbored nonpermissive infections, where parasites are restricted to the MG only (designated +/−), and those containing permissive infections, where trypanosomes are present in both the MG and the SGs (designated +/+). As a proxy for *W. morsitans* folate biosynthesis, the transcript abundances of folate biosynthesis genes (i.e., *aroA*, *aroC*, *pabB*, and *folP*) were compared between these two groups of flies exhibiting these two distinct infection states. Expression levels of *W. morsitans* folate biosynthetic loci were not significantly different in flies harboring +/− or +/+ *T. b. brucei* RUMP 503 infections (Fig. 2C). Furthermore, we were also interested in examining whether trypanosome-challenged tsetse flies maintained on glyphosate supplementation throughout the EIP would also be impacted in SG infection rates. Interestingly, MG-established *T. b. brucei* parasites were similarly capable of infecting the SGs of wild-type and glyphosate-supplemented *G. morsitans* (Fig. 2D) (*P* = 0.3261). Thus, folic acid appears to modulate the ability of this trypanosome to establish infections in *G. morsitans*’ MG but not its ability to migrate to and infect the fly’s SGs. This result corroborates those described above (Fig. 1), which show that *W. morsitans* folate biosynthesis gene transcript abundance is higher during early stages of the infection process.

These results confirm that *W. morsitans-*generated folate enhances the ability of distinct African trypanosomes to establish an initial MG infection in their tsetse vector. In contrast, the disruption of *W. morsitans* folate biosynthesis does not inhibit the MG-established trypanosomes from migrating to and infecting the tsetse’s SGs. This finding indicates that trypanosomes present different metabolic requirements during distinct stages of their life cycle in the tsetse vector. This finding aligns with previous studies revealing differential expression of *T. b. brucei* genes during development in tsetse tissues, at both the global level (41) and a more targeted level (42, 43).

### Vector competency of different tsetse species correlates with *Wigglesworthia* folate production.

**FIG 3:**
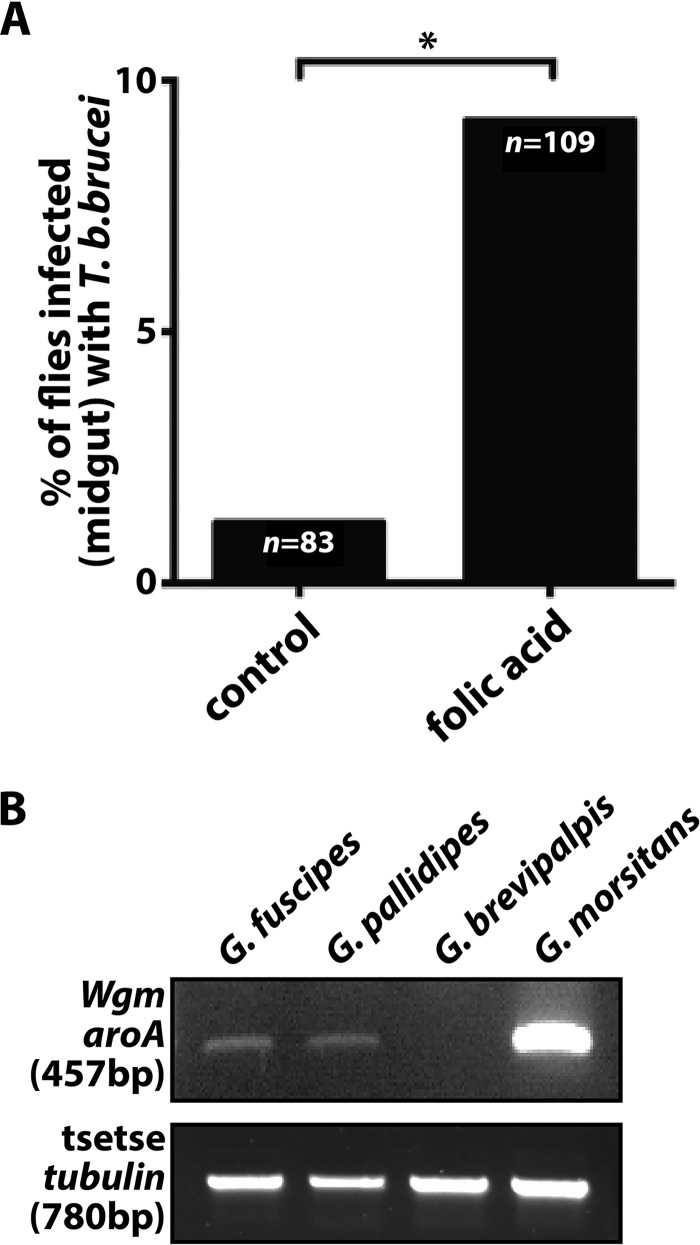
**(**A) Absolute MG infection rates (percent) in 2-week-old *G. brevipalpis* flies challenged with *T. b. brucei* RUMP 503. Control, flies maintained on blood only; folic acid, flies maintained on blood plus 500 nM folic acid. The means of data from two independent trials are indicated. Samples sizes (*n*) are provided. *, *P ≤ *0.05. (B) The presence of the *Wigglesworthia aroA* gene was assessed using 100 ng of total bacteriome DNA from *G. fuscipes*, *G. pallidipes*, *G. brevipalpis*, and *G. morsitans* and *W. morsitans aroA* primers. Amplification of the *Glossina* beta-tubulin gene was used as a control for DNA quality.

We discovered that *W. morsitans-*derived folate is a mediator of trypanosome infection outcomes in the MG of *G. morsitans*. We next employed the Glossina brevipalpis system to further validate the functional correlation between symbiont-derived folate and the ability of trypanosomes to establish an infection in the MG of their tsetse vector. The species G. brevipalpis is recognized as an inefficient vector of African trypanosomes (27–31), and this phenotype could result from the intersection of several genetic and ecological factors. One such factor may be the inability of Wigglesworthia brevipalpis to produce folate (Fig. S1), which is a valid theory considering our discovery that this vitamin facilitates trypanosome infection establishment in *G. morsitans*. To test this theory, we maintained *T. b. brucei*-challenged *G. brevipalpis* flies on a diet supplemented with 500 nM folic acid for 2 weeks and then monitored MG infection prevalence. We observed that significantly more *G. brevipalpis* flies receiving folic acid supplementation housed MG infections than did age-matched controls (9.2% versus 1.2%, respectively) (Fig. 3) that were fed normal blood. Thus, the absence of *W. brevipalpis-*generated folate is one factor that contributes to the low trypanosome infection prevalence observed in *G. brevipalpis* populations. As such, this vitamin may account in part for the differential vector competencies exhibited by distinct tsetse species. Finally, the detection of the *aroA* gene within *Wigglesworthia* isolates harbored by two other medically relevant tsetse species, Glossina pallidipes (44) and Glossina fuscipes (45), supports the presence of a folate biosynthesis capacity of these isolates and the likely importance of symbiont resource provisioning toward host vector competence (Fig. 3B).

## DISCUSSION

An important role of mutualistic bacteria is the provisioning of a reliable flow of nutrients to their host. When a disruption of the microbiota structure and/or function occurs (a phenomenon known as dysbiosis), host physiological perturbations arise, which can significantly reduce fitness (46–48). It remains unknown whether microbial dysbiosis also detrimentally impacts the fitness of parasitic organisms. Here we use the tsetse fly model system to characterize a novel functional relationship between metabolites produced by an ancient obligate symbiont, *Wigglesworthia*, and the ability of a parasite (T. brucei subspp.) to successfully infect its insect vector. Although multiple instances of manipulation of host metabolism aimed at prolonging infection and/or enhancing transmission have been demonstrated by pathogens such as Plasmodium falciparum, Trypanosoma cruzi, and Bordetella pertussis (reviewed in reference 49), this study is the first to report parasite exploitation of metabolic products produced by a disease vector’s bacterial symbiont. Specifically, we report that a specific symbiont-generated metabolite (vitamin B_9_) impacts insect vector competence by facilitating parasite infection establishment and, thus, subsequent transmission. This parasite dependence on a symbiont-derived vitamin represents a relationship that may be exploited to reduce disease transmission.

Different tsetse species exhibit distinct vector competencies (reviewed in reference 39), and here we provide evidence that these phenotypes are in part associated with the concomitant ability of mutualistic *Wigglesworthia* bacteria to synthesize folate in their fly host. In support of this finding, we report that the exogenous provisioning of folate to tsetse that harbor *Wigglesworthia* incapable of synthesizing B_9_ (i.e., *G. brevipalpis*) increases the flies’ susceptibility to trypanosome infection, additionally implicating folate as a limited resource impacting vector competence. We hypothesize that *W. morsitans-*provisioned folate is hijacked by auxotrophic trypanosomes to facilitate their development within the tsetse’s MG, which comes at the expense of tsetse sexual maturation and reproduction (12). This theory is supported by the fact that infected tsetse flies exhibit reduced fecundity (50). The flip side of the metabolic tug-of-war between tsetse and trypanosomes is represented by the fact that typically ≤10% of wild flies are infected with trypanosomes (51), even though they feed regularly on vertebrate reservoirs. This low infection prevalence implies that symbiont-mediated enhancement of trypanosome infection establishment, the tsetse’s robust antiparasitic immune response (40, 52–55), and the nutritional demands of fly reproduction may all be competing physiological processes. These findings reflect the challenge that African trypanosomes face when competing with their tsetse host for exogenous resources that both organisms require for their survival.

We report that the expression of *Wigglesworthia* folate biosynthesis genes within the tsetse bacteriome, in addition to responding to tsetse age and reproduction (12), is also affected by the presence of trypanosomes. The greatest impact on the expression of folate biosynthesis genes occurred during the early infection stage (ES), when parasites are trying to colonize the MG. To further examine the impact of *W. morsitans* folate provisioning (and, correspondingly, its disruption) on the ability of trypanosomes to successfully infect tsetse, we experimentally manipulated the bacterium’s chorismate pathway, which feeds folate biosynthesis, by continuously supplementing *G. morsitans* blood meals with glyphosate. Glyphosate inhibits 5-enolpyruvylshikimate-3-phosphate synthase (EPSPS) (56), the enzyme encoded by the *aroA* gene, by preventing the incorporation of PEP and 3-phosphoshikimate, which is then converted to chorismate. The *W. morsitans aroA* locus phylogenetically clusters within the class I EPSPS enzymes that are found within eukaryotes, archaea, and some bacteria, which are glyphosate sensitive (see Fig. S4 in the supplemental material), in contrast to bacteria with class II EPSPS enzymes, which support greater glyphosate resilience (57). Glyphosate exposure leads to cell growth arrest but is not lethal to bacteria that contain class I EPSPS enzymes within their genomes (58) (Fig. S4). Although EPSPS is widely distributed among plants and microorganisms, homologs are lacking in tsetse (59) and African trypanosomes (22).

10.1128/mBio.00018-19.4 FIG S4 Phylogeny of class I and class II EPSPS enzymes. Amino acid sequences for the *aroA* genes were obtained from a previous study (E. V. S. Motta, K. Raymann, and N. A. Moran, Proc Natl Acad Sci U S A, 115:10305–10310, 2018, https://doi.org/10.1073/pnas.1803880115), with the exception of Wigglesworthia glossinidia (GenBank accession number WP_014354051.1), Sodalis praecaptivus (GenBank accession number WP_025422806.1), Sodalis glossinidius (GenBank accession number CRL44853.1), and “*Candidatus* Sodalis pierantonius” (GenBank accession number AHF73967.1). MUSCLE (R. C. Edgar, BMC Bioinformatics, 5:113, 2004, https://doi.org/10.1186/1471-2105-5-113) was used for sequence alignment. Molecular phylogenetic analysis was performed using maximum likelihood (LG model plus gamma, with 4,100 bootstrap replicates) with PhyML v3.1 (S. Guindon, J. F. Dufayard, V. Lefort, M. Anisimova, et al., Syst Biol 59:307–321, 2010, https://doi.org/10.1093/sysbio/syq010) with 100 bootstrap replicates in Seaview v4.7 (M. Gouy, S. Guindon, and O. Gascuel, Mol Biol Evol 27:221–224, 2010, https://doi.org/10.1093/molbev/msp259). Shaded circles indicate bootstrap support values. Download FIG S4, PDF file, 0.1 MB. Copyright © 2019 Rio et al. 2019 Rio et al. This content is distributed under the terms of the Creative Commons Attribution 4.0 International license.

Nutrient demands of cells fluctuate in response to environmental changes, including variations in pH, temperature, and carbon source availability, which is nicely exemplified by the comparison of trypanosome metabolism within the mammalian host versus its tsetse vector (60–63). In this study, glyphosate supplementation in tsetse fly blood meals significantly reduced trypanosome MG infection prevalence in *G. morsitans*, with this phenotype being rescued with the addition of exogenous folate. In contrast, glyphosate treatment had no effect on the ability of trypanosomes to migrate to and infect the tsetse’s SGs. These findings further emphasize that trypanosomes present different metabolic demands during vector-specific stages of their life cycles (41–43), similar to when these parasites occupy distinct niches within their mammalian hosts (i.e., adipose tissue versus the bloodstream) (64). Importantly, trypanosomes must establish an infection in the tsetse’s MG in order to progress to the fly’s SGs. Thus, despite the fact that similar proportions of glyphosate-treated and wild-type tsetse presented infected SGs, the absolute number of glyphosate-treated flies with parasite-colonized SGs was comparatively low because few of them housed prerequisite MG infections.

The taxonomic composition of the tsetse’s microbiota is fly population dependent. The tsetse’s microbial community consists of various combinations of indigenous endosymbiotic (all tsetse house *Wigglesworthia*, but *Sodalis* and *Wolbachia* are absent from some individuals) and environmentally acquired (transient) enteric bacteria (8, 65). This microbial consortium makes tsetse an efficacious model system for studying metabolic interdependencies among members of a taxonomically divergent holobiont. A good example of this interdependency involves the tsetse’s secondary symbiont *Sodalis*, which is unable to produce thiamine (vitamin B_1_) but requires this vitamin in order to replicate in and infect insect cells. To meet this metabolic necessity, *Sodalis* scavenges *Wigglesworthia*-derived thiamine from its environment (66). The *Sodalis* genome also encodes all of the molecular machinery necessary to produce folate (67), with its EPSPS gene also clustering within class I (Fig. S4). Furthermore, *Sodalis* prevalence and/or density positively correlates with trypanosome infection outcomes (65, 68, 69). Based on our findings that folate facilitates trypanosome infection outcomes in tsetse, the role of *Sodalis* in enhancing parasite infectivity may include the bacterium’s ability to export additional quantities of the vitamin to the fly’s MG, where trypanosomes use it to establish an infection. Additional studies are warranted to determine the metabolic relationship between tsetse, its environmentally acquired enteric bacteria, and the other members of the fly’s microbiota.

Vector competence is determined by an integrated, multifactorial set of intrinsic factors as well as extragenetic, ecological variables. In the case of tsetse, genetic factors, including the production of antimicrobial peptides and reactive oxygen species, in conjunction with ecological factors, such as fly age, feeding status, and microbiota composition, all impact the proclivity of tsetse toward trypanosome infection (reviewed in references 70–74). Importantly, we have moved away from examining these traits in isolation and are instead developing an appreciation for their overlap and interconnectedness as phenotypic determinants. For example, we demonstrate that *Wigglesworthia* provides support to trypanosomes by providing an essential nutrient that the parasite cannot generate on its own. Conversely, this bacterium also supports the ability of tsetse to clear fitness-reducing trypanosome infections by actuating the development and function of the fly’s immune system. Specifically, tsetse larvae that mature in the absence of *Wigglesworthia* are severely immunocompromised as adults. This phenotype is characterized by a reduced population of phagocytic hemocytes, atypical expression of various immunity-related genes, and a structurally compromised peritrophic matrix (75–77). These immunostimulatory phenotypes may be mediated by one or a combination of *Wigglesworthia*-derived factors, including vitamins and other nutrients such as secondary metabolites. Multiple variables are known to influence immune function, including nutritional status (78). More specifically, several micronutrients and trace elements are essential determinants of epithelial barrier strength and the responsiveness of cellular and humoral immune factors (79). Thus, the *Wigglesworthia* symbiont represents an extragenetic, yet heritable, factor that impacts trypanosome infection outcomes by contributing to the fitness of both the parasite and the vector host.

Trypanosomes must have access to folate in order to infect vertebrate hosts, and this requirement could be exploited to develop novel chemotherapeutic approaches for treating trypanosomiases (33, 80–82). However, prophylactic measures are favorable alternatives to treating already sick patients, and as such, inhibiting the ability of pathogens and parasites to establish an infection in their arthropod vector would prove epidemiologically beneficial. Along this line, our finding that trypanosome infection establishment in tsetse is impacted by the presence of symbiont-generated folate represents an obligate physiological dependency that may be interrupted to reduce tsetse vector competency. Furthermore, disrupting this symbiont vitamin-provisioning pathway also decreases tsetse fly fitness (12), thereby controlling trypanosome transmission in a multifaceted manner. Similarly, host-symbiont-pathogen metabolic liaisons that occur in other arthropod vectors, as well as in more complex vertebrate model systems, may also be exploited to reduce disease, offering new avenues to tap into for advancements in health and medicine.

## MATERIALS AND METHODS

### Ethical consideration.

Trypanosome infection assays were performed at the Yale School of Public Health. This work was carried out in strict accordance with the recommendations of the Office of Laboratory Animal Welfare of the National Institutes of Health and the Yale University Institutional Animal Care and Use Committee (IACUC). The experimental protocol was reviewed and approved by the Yale University IACUC (protocol 2011-07266).

### Tsetse and trypanosomes.

The tsetse species Glossina morsitans and *G. brevipalpis* were maintained in Yale’s insectary at 24°C with 50 to 55% relative humidity on a 12-h*-*light/12*-*h*-*dark photoperiod schedule. All flies received defibrinated bovine blood (Hemostat Labs) every 48 h through an artificial membrane feeding system (83). Colony flies harbor *Wigglesworthia*, *Sodalis*, and *Wolbachia* infections. All tsetse female flies used were unmated.

Trypanosoma brucei *brucei* strain RUMP 503 and *T. b. rhodesiense* strain YTat 1.1 were multiplied in rats and harvested from infected blood at peak parasitemia. Blood was aliquoted and cryopreserved for subsequent tsetse challenge experiments. *T. b. brucei* RUMP 503 completes its entire developmental cycle in tsetse and was thus used to initiate MG and SG infections. YTat 1.1 cells are restricted to establishment of MG infections, as this parasite line is unable to establish SG infections in tsetse (50).

### Expression analyses of *W. morsitans* B vitamin biosynthesis loci.

To examine the expression of *Wigglesworthia* (*G. morsitans* isolate referred to here as “*W. morsitans*”) genes involved in folate biosynthesis during trypanosome challenge, teneral tsetse flies received an initial blood meal that contained bloodstream *T. b. brucei* RUMP 503 parasites (1 × 10^6^ parasites/ml). Challenged flies were dissected 2 or 5 weeks later to score early-stage (ES) MG and late-stage (LS) MG and SG infection prevalences, respectively. Flies that had cleared their infections were designated “parasite refractory” (PR). Bacteriomes were dissected, and RNA was isolated from infected ES and LS PR flies and sex- and age-matched nonchallenged (NC) controls according to the TRIzol protocol (Invitrogen). Total RNA was treated with DNase I (Ambion) and verified to be free of DNA contamination via PCR. The RNA concentration was measured using a Nanodrop spectrophotometer, and cDNA was synthesized from ∼140 ng RNA using a 2 μM primer cocktail of gene-specific 3′-end primers for *aroA* (3-phosphoshikimate 1-carboxyvinyltransferase), *pabB* (aminodeoxychorismate synthase subunit I), *folP* (7,8-dihydropteroate synthase) (primer sequences are provided in reference 12), and SuperScript II reverse transcriptase (Invitrogen).

Quantitative reverse transcription-PCR (RT-PCR) was used to analyze the expression of the representative *W. morsitans* chorismate and folate metabolic genes within fly bacteriomes after trypanosome challenge using the primers and protocol described previously (12). The constitutively expressed *Wigglesworthia* small-subunit ribosomal protein S3 (*rpsC*) (primer sequences are provided in reference 12) was used to normalize transcript levels. At least four independently acquired biological replicates were analyzed per treatment for each gene, with each sample replicated three times. Negative controls (i.e., water only, lacking a nucleic acid template) were included for all amplification reactions.

Quantitative RT-PCR was also used to assess the effect of glyphosate treatment on *W. morsitans* density and *thiC* expression (involved in B_1_ biosynthesis). Flies were fed 100 μM glyphosate in their first meal, with bacteriomes dissected 72 h later. Flies at this time point (4 days old) have actively replicating *Wigglesworthia* populations (66). Primer sequences for *Wigglesworthia thiC* were forward primer 5′-AAGTTATGATAGAAGGACCAGGAC-3′ and reverse primer 5′-CCCGGAGCAATATCAGTAGTTAG-3′. The *Wigglesworthia gapdh* (glyceraldehyde-3-phosphate dehydrogenase) gene (forward primer 5′-CTGATTTCGTTGGTGATACT-3′ and reverse primer 5′-CCAAATTCGTTGTCGTACCA-3′) was used for normalization. Six independently acquired biological replicates were analyzed per treatment for each gene, with each sample replicated three times. Negative controls (i.e., water only, lacking a nucleic acid template) were included for all amplification reactions.

### Impact of glyphosate on trypanosome duplication.

Procyclic-form *T. b. brucei* RUMP 503 parasites (1 × 10^6^ cells/ml) were incubated at 25°C to 28°C in Beck’s medium plus glyphosate (Sigma) at concentrations ranging from 10 mM to 10 μM. Parasite densities were determined by microscopy with the use of a Neubauer hemocytometer. The duplication rate was determined by taking the density at 48 h and normalizing this value to the starting density of the trypanosome culture (i.e., time point zero).

### Folate and trypanosome infection outcomes.

*G. morsitans* and *G. brevipalpis* were challenged *per os* with 1 × 10^6^ parasites/ml of bloodstream-form *T. b. brucei* RUMP 503 in their first blood meal. Identical challenges with *T. b. rhodesiense* YTat 1.1 were also performed with *G. morsitans.* In the case of *G. morsitans*, the antioxidant cysteine (10 μM) was added to the infective blood meal to increase trypanosome infection prevalence (40). Subsequently, all flies were fed defibrinated bovine blood (Hemostat Labs, CA) every other day, with control individuals receiving normal blood and treatment individuals receiving blood meals containing either a folate inhibitor and/or folate supplementation. Specifically, distinct treatment groups of *G. morsitans* flies were maintained on a diet containing 100 μM glyphosate [*N*-(phosphonomethyl)glycine; Sigma] or 100 μM glyphosate combined with 500 nM folic acid (Sigma). Conversely, *G. brevipalpis* flies were fed blood supplemented with 500 nM folic acid as a means of complementing the dysfunctional folate biosynthesis pathway presented by the native *Wigglesworthia* isolate.

At 2 and 5 weeks postchallenge, *G. morsitans* MGs and MGs and SGs, respectively, were dissected and microscopically scored for the presence or absence of trypanosomes. At 14 days postchallenge, *G. brevipalpis* MGs were dissected and microscopically scored for the presence or absence of trypanosomes.

### Occurrence of *aroA* within *Wigglesworthia* isolates.

The presence of the *Wigglesworthia aroA* gene was assessed using 100 ng of total bacteriome DNA isolated from G. fuscipes, G. pallidipes, *G. brevipalpis*, and *G. morsitans.* The *W. morsitans aroA* primers used were forward primer 5′-TTT TAT TAT CGG CGC AAA CC-3′ and reverse primer 5′-AAT GGG GCC ATG ATG AGT AA-3′, with an annealing temperature (*T_a_*) of 55°C, and resulted in the amplification of a 450-bp product using *G. morsitans* bacteriome DNA as the template. The tsetse beta-tubulin gene was used as a control for DNA quality (forward primer 5′-ACGTATTCATTTCCCTTTGG-3′ and reverse primer 5′-AATGGCTGTGGTGTTGGACAAC-3′; *T_a_* of 55°C; 780-bp amplicon).

### Statistical analyses.

To compare *W. morsitans* gene expression levels following trypanosome challenge, all data were subjected to a goodness-of-fit test to determine the normality of distributions. One-way analysis of variance (ANOVA) and Tukey-Kramer *post hoc* pairwise comparisons of the means were performed using JMP 7.0 (SAS Institute).

For examining the prevalence of trypanosome infections within challenged flies, statistical analyses were carried out using R software for macOS (version 3.3.2). A generalized linear model (GLM) was generated using binomial distribution with a logit transformation of the data. For the glyphosate inhibition experiments, either the binary infection status (recovered or infected) or the binary type of infection (MG only or MG and SGs) was analyzed as a function of the treatment that the flies received (control, glyphosate, or glyphosate plus folate) and the replicate to which it belonged. For the folate supplementation experiment in *G. morsitans*, the binary infection status (recovered or infected) was analyzed as a function of the treatment that the flies received (control or folate). For the folate supplementation experiment in *G. brevipalpis*, the binary infection status (recovered or infected) was analyzed as a function of the treatment that the flies received (control or folate) and the sex of the flies (male or female). The best statistical model was searched using a backward stepwise procedure from the full additive model testing the main effect of each categorical explanatory factor. Using the retained models, we performed Wald tests on the individual regression parameters to test their statistical difference from the control.
