# Supplementary material for: Mutualist-Provisioned Resources Impact Vector Competency
Source: mBio. 2019 Jun 4;10(3):e00018-19. doi: 10.1128/mBio.00018-19 (PMC6550517; doi:10.1128/mBio.00018-19)
Supplement: FIG S2 [file mBio.00018-19-sf002.pdf]

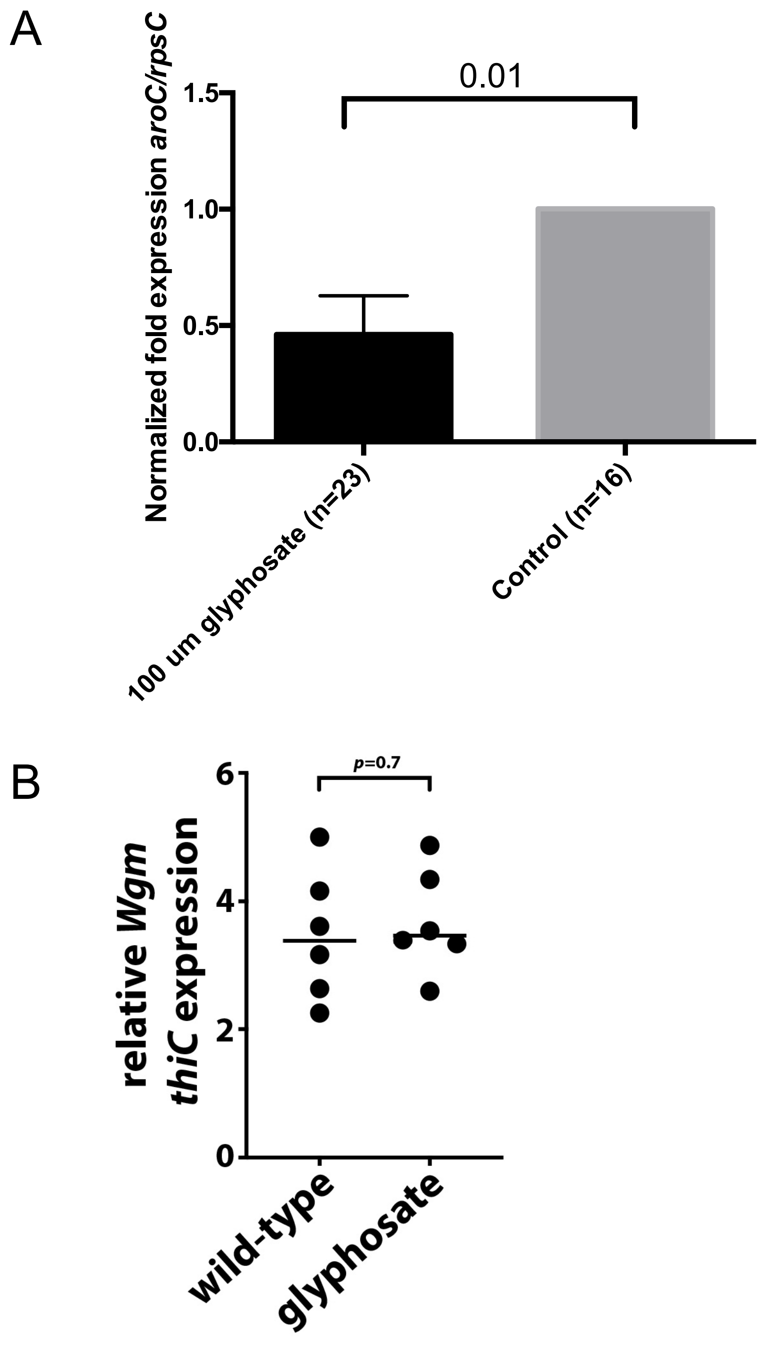

Supplemental Figure 2. **A.** The expression of *Wgm aroC*, which catalyzes the seventh step in chorismate production, within bacteriomes following glyphosate supplementation of tsetse blood meals. The expression of *Wgm aroC,* located immediately downstream of *aroA*, significantly decreases with glyphosate supplementation of blood meals relative to control. The *Wgm rpsC* was used as a reference gene for normalization. The expression of *aroC*/*rpsC* in untreated age-matched tsetse was set as 1. Sample sizes (*n*) are indicated. **B.** *Wgm* titers and *thiC* expression (involved in B1 synthesis) remain unaffected following glyphosate treatment. Each dot represents *Wgm* expression within the bacteriome of an individual fly.
