# Supplementary material for: Mutualist-Provisioned Resources Impact Vector Competency
Source: mBio. 2019 Jun 4;10(3):e00018-19. doi: 10.1128/mBio.00018-19 (PMC6550517; doi:10.1128/mBio.00018-19)
Supplement: FIG S3 [file mBio.00018-19-sf003.pdf]

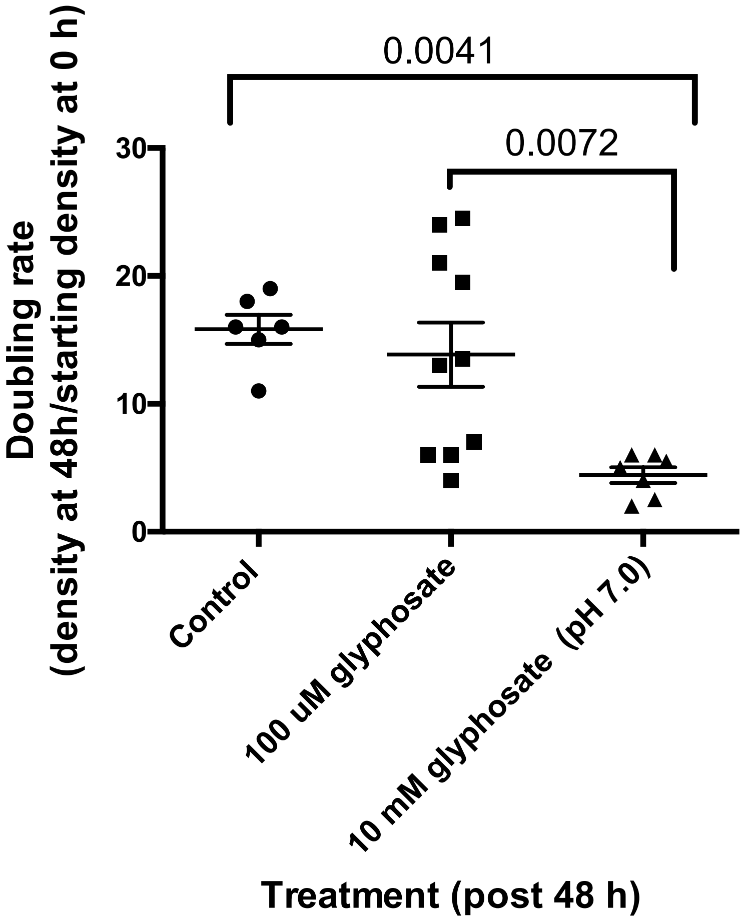


Supplemental FIG. 3. Bloodstream *T.b. brucei* RUMP 503 doubling rate ± [glyphosate] within *in vitro* culture. With 100 uM glyphosate supplementation neither trypanosome growth nor morphology were affected. The mean and standard error of the mean (SEM) of two independent trials is demonstrated.
