# Supplementary material for: Mutualist-Provisioned Resources Impact Vector Competency
Source: mBio. 2019 Jun 4;10(3):e00018-19. doi: 10.1128/mBio.00018-19 (PMC6550517; doi:10.1128/mBio.00018-19)
Supplement: FIG S4 [file mBio.00018-19-sf004.pdf]

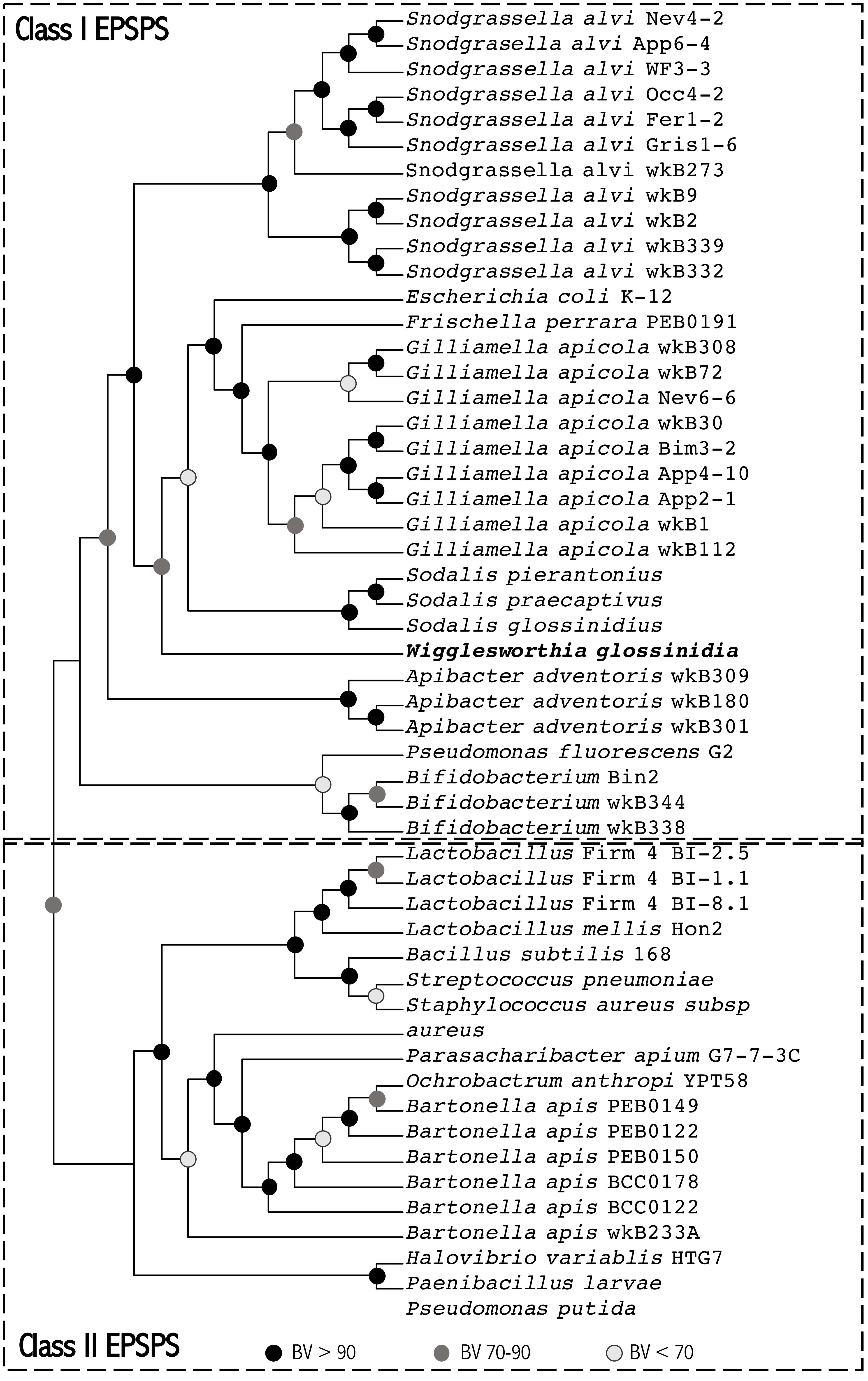
Class I EPSPS
Snodgrasella alvi Nev4-2
Snodgrasella alvi App6-4
Snodgrasella alvi WF3-3
Snodgrasella alvi Occ4-2
Snodgrasella alvi Fer1-2
Snodgrasella alvi Gris1-6
Snodgrasella alvi wkB273
Snodgrasella alvi wkB9
Snodgrasella alvi wkB2
Snodgrasella alvi wkB339
Snodgrasella alvi wkB332
Escherichia coli K-12
Frischella perrara PEB0191
Gilliamella apicola wkB308
Gilliamella apicola wkB72
Gilliamella apicola Nev6-6
Gilliamella apicola wkB30
Gilliamella apicola Bim3-2
Gilliamella apicola App4-10
Gilliamella apicola App2-1
Gilliamella apicola wkB1
Gilliamella apicola wkB112
Sodalis pierantonius
Sodalis praecaptivus
Sodalis glossinidius
Wigglesworthia glossinidia
Apibacter adventoris wkB309
Apibacter adventoris wkB180
Apibacter adventoris wkB301
Pseudomonas fluorescens G2
Bifidobacterium Bin2
Bifidobacterium wkB344
Bifidobacterium wkB338
Lactobacillus Firm 4 BI-2.5
Lactobacillus Firm 4 BI-1.1
Lactobacillus Firm 4 BI-8.1
Lactobacillus mellis Hon2
Bacillus subtilis 168
Streptococcus pneumoniae
Staphylococcus aureus subsp
aureus
Parasacharibacter apium G7-7-3C
Ochrobactrum anthropi YPT58
Bartonella apis PEB0149
Bartonella apis PEB0122
Bartonella apis PEB0150
Bartonella apis BCC0178
Bartonella apis BCC0122
Bartonella apis wkB233A
Halovibrio variablis HTG7
Paenibacillus larvae
Pseudomonas putida
Class II EPSPS
BV > 90
BV 70-90
BV < 70

Supplemental Figure 4. Phylogeny of class I and class II EPSPS enzymes. Amino acid sequences for the *aroA* genes were obtained from [1] with the exception of *Wigglesworthia glossinidia* (WP_014354051.1), *Sodalis praecaptivus* (WP_025422806.1), *S. glossinidius* (CRL44853.1) and "*Candidatus* Sodalis pierantonius" (AHF73967.1). MUSCLE [2] was used for sequence alignment. Molecular phylogenetic analysis was performed using maximum-likelihood (LG Model + Gamma4, 100 bootstrap replicates) with PhyML v3.1 [3] with 100 bootstrap replicates in Seaview v4.7 [4]. Shaded circles indicate bootstrap support values.

[1] Motta, Erick V.S., *et al.* Glyphosate perturbs the gut microbiota of honey bees. *PNAS*. 2018 115(41):10205-10310.

[2] Edgar, R.C. MUSCLE: A multiple sequence alignment method with reduced time and space complexity. *BMC Bioinformatics* 2004 5:113.

[3] Guindon S. *et al.* Algorithms and Methods to Estimate Maximum-Likelihood Phylogenies: Assessing the Performance of PhyML 3.0. *Systematic Biology* 2010, 59(3):307-21.

[4] Guoy, M. *et al.* SeaView version 4: A multiplatform graphical user interface for sequence alignment and phylogenetic tree building. *Molecular Biol. Evol.* 2010. 27(2): 221-4.
