# Supplementary material for: Mutualist-Provisioned Resources Impact Vector Competency
Source: mBio. 2019 Jun 4;10(3):e00018-19. doi: 10.1128/mBio.00018-19 (PMC6550517; doi:10.1128/mBio.00018-19)

Supplemental Figure 1: The *Wigglesworthia* isolate harbored within *G. morsitans* (*Wgm)* retains the complete chorismate and folate biosynthesis pathway. The sequential chorismate and folate production pathways are represented by arrows indicating steps catalyzed by the named enzymes. The initial molecule phosphoenolpyruvate (PEP) is a byproduct of glycolysis, while erythrose-4-phosphate is an intermediate in the pentose phosphate pathway. The enzymatic inhibition of glyphosate towards AroA is shown.

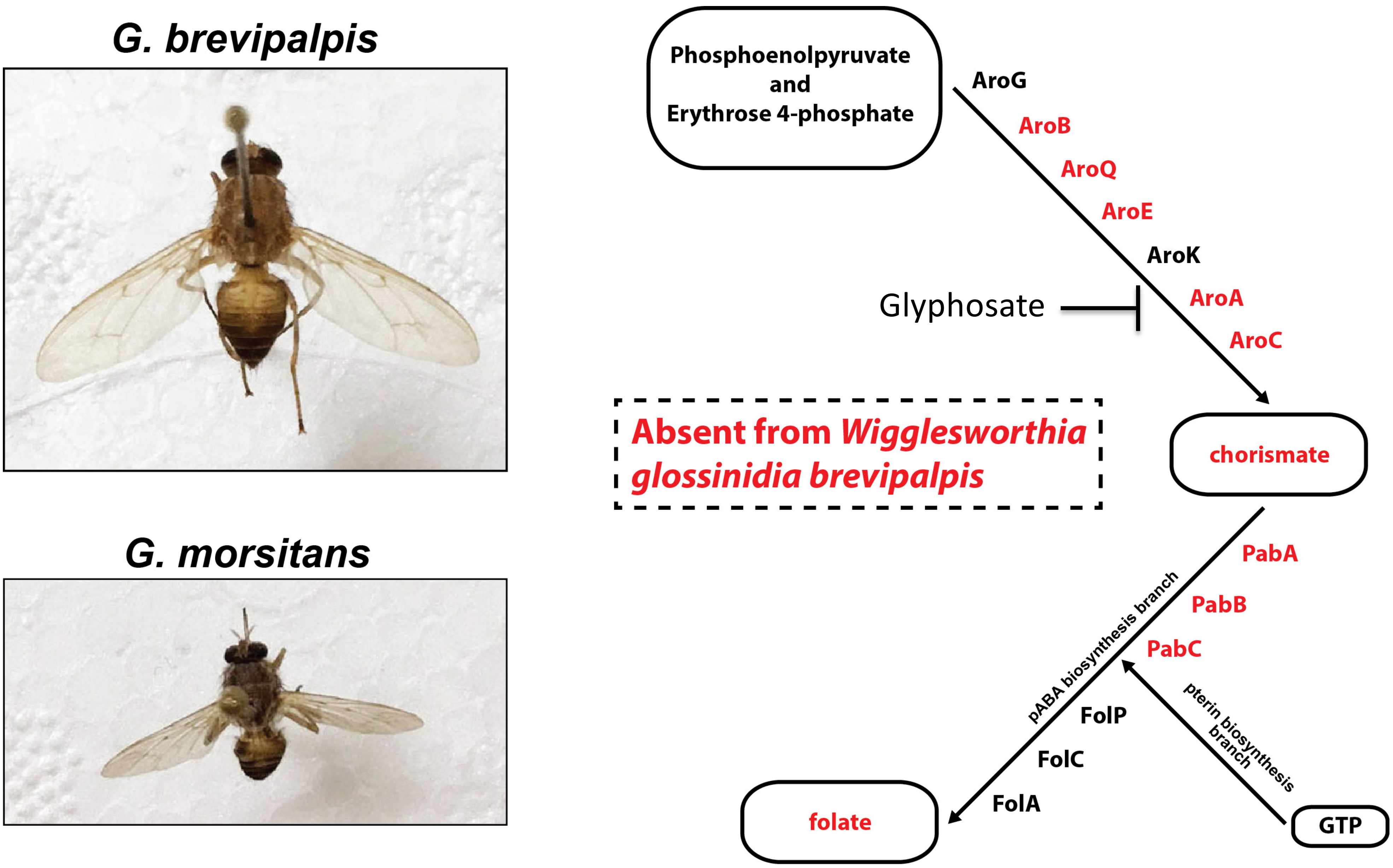

Supplement: FIG S1 [file mBio.00018-19-sf001.pdf]
